# Role of extracellular ATP and P2 receptor signaling in regulating renal cyst growth and interstitial inflammation in polycystic kidney disease

**DOI:** 10.3389/fphys.2013.00218

**Published:** 2013-08-16

**Authors:** Gopi Rangan

**Affiliations:** Michael Stern Translational Laboratory for Polycystic Kidney Disease, Centre for Transplant and Renal Research, Westmead Millennium Institute, University of SydneySydney, NSW, Australia

**Keywords:** polycystic kidney disease, cyst, interstitial inflammation, purinergic, ATP

## Abstract

Polycystic kidney diseases (PKD) are a group of inherited ciliopathies in which the formation and growth of multiple cysts derived from the distal nephron and collecting duct leads to the disruption of normal kidney architecture, chronic interstitial inflammation/fibrosis and hypertension. Kidney failure is the most life-threatening complication of PKD, and is the consequence of cyst expansion, renal interstitial disease and loss of normal kidney tissue. Over the last decade, accumulating evidence suggests that the autocrine and paracrine effects of ATP (through its receptor family P2X and P2Y), could be detrimental for the progression of PKD. (2009). *In vitro*, ATP-P2 signaling promotes cystic epithelial cell proliferation, chloride-driven fluid secretion and apoptosis. Furthermore, dysfunction of the polycystin signal transduction pathways promotes the secretagogue activity of extracellular ATP by activating a calcium-activated chloride channel *via* purinergic receptors. Finally, ATP is a danger signal and could potentially contribute to interstitial inflammation associated with PKD. These data suggest that ATP-P2 signaling worsens the progression of cyst enlargement and interstitial inflammation in PKD.

## Introduction

Polycystic kidney diseases (PKD) are a group of inherited ciliopathies in which the formation and growth of multiple fluid-filled renal cysts leads to disruption of normal kidney architecture, chronic renal interstitial inflammation and fibrosis, and hypertension (Harris and Torres, 2009). Kidney failure is the most serious and life-threatening complication of PKD (Rangan et al., 2005), and is the consequence of cyst expansion, renal interstitial disease and loss of normal kidney tissue (Grantham et al., 2011). Cyst growth in PKD is due to a combination of proliferation of cyst-lining epithelial cells of the distal nephron and collecting ducts, chloride-driven fluid secretion and extracellular matrix defects (Grantham et al., 1989). Although there is no treatment to currently prevent kidney failure due to PKD, rapid progress is being made (Harris and Torres, 2009). For example, in 2012, a 3-year Phase 3 clinical trial using a small molecule inhibitor (tolvaptan, a vasopressin type 2 receptor antagonist) successfully attenuated the rate of kidney enlargement in humans with PKD (Kelsey, 2013). The premise for the use of vasopressin receptor antagonism was based on preclinical data showing that vasopressin altered in intracellular purinergic (cAMP) signaling in PKD (Wang et al., 2005).

It has been hypothesized that a multi-drug approach, targeting cyst growth and interstitial inflammation, will be needed to effectively prevent kidney failure in PKD (Aguiari et al., 2013), and therefore further therapies are needed (Chang and Ong, 2013). For over a decade, it has been postulated that the autocrine and paracrine effects of extracellular nucleotides and their metabolites could be detrimental for the progression of PKD, and that strategies to attenuate this activation using inhibitors of nucleotide release, nucleotide scavengers or nucleotide antagonists could be useful for the therapy of PKD (Wilson et al., 1999; Schwiebert, 2001; Leipziger, 2003; Hillman et al., 2005; Hovater et al., 2008; Xu et al., 2009). Currently, knowledge regarding the role of extracellular nucleotides in the pathogenesis of kidney failure due to PKD remains in the early phase of clinical translation. This is highlighted by the paucity of published articles, as a PubMed search performed on the 29th April 2013, using the terms “ATP and polycystic kidney disease,” revealed 42 publications, only 15 of which were original scientific articles and 6 were review articles that were directly relevant to this field of research. Nevertheless, significant insights have been gained from this limited information to support that extracellular nucleotide signaling is worthy of further pursuit in PKD. In fact, it has been hypothesized that this approach might act synergistically with vasopressin receptor antagonism (Buchholz et al., 2011; Luft, 2011). Extracellular nucleotides and metabolites can be classified according to the base from which they are derived and the number of associated number of phosphate groups (adenosine-derived; ATP, ADP; AMP; guanosine-derived: GTP, GDP, GMP; cytidine-derived: CTP, CDP, CMP; 5-methyluridine- and uridine-derived). The majority of the available data regarding extracellular nucleotides in PKD is based on ATP-P2 receptor signaling. Therefore, this mini-review will focus primarily on the role of ATP and its P2 receptors, in the pathogenesis and progression of cyst enlargement and interstitial inflammation in PKD.

## Overview of PKD

PKD consists of several variants which differ according to their mode of inheritance, underlying genetic mutation and pattern of cyst formation. These variants include automosal dominant PKD (ADPKD), autosomal recessive PKD (ARPKD) and other autosomal recessive cystic renal diseases, including nephronophthisis (NPHP). ADPKD is the most common type of cystic renal disease, with an incidence of approximately one in every 1000 live births, and estimated to affect 6.5 million people world-wide (Bisceglia et al., 2006; Harris and Torres, 2009). Structurally, in ADPKD, the cysts are spherical in shape and occur in both kidneys. Cyst growth is not synchronized. Therefore the end-stage kidney in ADPKD contains thousands of cysts that vary in size from 100 μm to several centimeters in diameter, resulting in a large (weighing up to 3–5 kg, compared to 125–170 g in normal males) and irregularly shaped organ. In ADPKD, the cysts start to form in early life (possibly *in utero* or during the early postnatal period) due to the clonal proliferation of focal epithelial cells lining the distal nephron and collecting duct, which leads to diverticular-like protrusions extending into interstitium (Baert, 1978). With continued growth, the latter detach from the parent nephron when their diameter exceeds 100 μm, and form “encapsulated cysts” (Hovater et al., 2008). Once in the interstitium the cysts continue to slowly expand over many decades (Grantham et al., 2011). Kidney failure therefore occurs after a long latent period, usually by the 5th decade of life (Baert, 1978), when a sufficient number of cysts (possibly > 1000) (Luft, 2011) have collectively grown to disrupt normal kidney architecture and function. In contrast, ARPKD is a less frequent, childhood disease (1:20 000 live births) (Sweeney and Avner, 2006). It is characterized by the synchronized microcystic dilation of collecting ducts. Detachment of the dilated cystic collecting ducts from the nephron does not occur (Baxter, 1961). The kidneys are large but maintain their reniform shape, and kidney failure typically occurs during the neonatal period (Sweeney and Avner, 2006). NPHP has an autosomal recessive mode of inheritance, and is characterized by tubulointerstitial nephropathy and corticomedullary duct ectasia, but kidney enlargement does not occur (Wolf and Hildebrandt, 2011). NPHP is one of the most frequent genetic disorders causing kidney failure in children and adolescents (Wolf and Hildebrandt, 2011).

ADPKD, ARPKD and NPHP are caused by mutations in the *Pkd1/Pkd2, Pkhd1* and *Nphp* (Grantham et al., 1989, 2011; Wilson et al., 1999; Schwiebert, 2001; Rangan et al., 2005; Wang et al., 2005; Harris and Torres, 2009; Xu et al., 2009; Aguiari et al., 2013; Chang and Ong, 2013; Kelsey, 2013) genes respectively, which encode the proteins, polycystin (PC)-1/PC-2, fibrocystin and nephrocystin (Sweeney and Avner, 2006; Harris and Torres, 2009). These so-called “cystproteins” (Hildebrandt and Otto, 2005) have all been found to co-localize to the primary renal cilia (an antenna-like sensory organelle involved in mechanosensation), and interact with each other at a molecular and functional level (Kaimori and Germino, 2008; Fedeles et al., 2011). In physiological states, the intact cystoprotein complex maintains the normal function of the cilium, negatively regulates the cell-cycle (Bhunia et al., 2002) and promotes intracellular calcium transport (Cowley, 2008) and cellular differentiation as well as normal renal tubular morphogenesis (Boletta and Germino, 2003). The intracellular level of PC-1 plays a central role in both ADPKD and ARPKD, as it is the rate-limiting component that ultimately determines cyst formation (Fedeles et al., 2011). Interestingly, ADPKD is a focal disease, as only 1–2% of nephrons in a kidney develop cysts (Martinez and Grantham, 1995). It has therefore been postulated that a heterozygote germ-line mutation in *Pkd1* or *Pkd2*, combined with postnatal disruption of the second normal allele is required for cyst formation (the two-hit mechanism of the Knudson theory) (Nauli et al., 2006). An additional molecular explanation for the postnatal onset of ADPKD is an age-related decline in the dosage of functional PC-1 protein (Rossetti et al., 2009). In contrast to ADPKD, postnatal somatic mutations do not have a role in cyst formation in ARPKD (Sweeney and Avner, 2006). Despite their importance in PKD, therapeutic approaches to modulate the genetic expression of PCs has been problematic because: (1) there is significant heterogeneity in the genetic mutations of PC-1 (Harris and Torres, 2009); (2) over- as well as under-expression of PC-1 can cause PKD (Harris and Torres, 2009); (3) there are difficulties with the delivery of viral vectors for gene therapy due to the poor endocytosis capability of the epithelial cells lining cysts (Witzgall et al., 2002). Therefore, treatments to prevent kidney failure due to PKD have focused on the abnormalities in cellular function and signal transduction arising from the dysfunction of cystproteins (Torres et al., 2007). These abnormalities are largely a consequence of reduced intracellular calcium which leads to increased intracellular levels of cAMP. As discussed earlier, cAMP levels are also increased by circulating vasopressin.

Halting the growth of cysts is one of the main therapeutic goals in preventing the decline of kidney function in PKD. The mechanisms of cyst growth involve three factors (Torres et al., 2007; Terryn et al., 2011): (i) increased proliferation of CECs: (ii) the gradual and incremental accumulation of fluid inside cysts; and (iii) abnormalities in extracellular matrix formation and degradation which are permissive for cyst expansion. The accumulation of fluid in cysts is an important mechanism of disease progression in PKD, and as discussed below, may be induced by purinergic signaling (Terryn et al., 2011). It is due to the net transepithelial secretion of chloride across apical membranes of CECs *via* chloride channels [cystic fibrosis transmembrane conductance regulator in response to the induction of cAMP-mediated protein kinase A (CFTR-PKA); and calcium-activated chloride channels]. This chloride efflux then induces Na+ (as a result electric coupling) and water (as a result of osmotic coupling via aquaporin channels) efflux, resulting in the progressive accumulation of fluid within the cysts (Terryn et al., 2011).

## Potential mechanisms of extracellular ATP release and P2 receptor signaling in PKD

The intracellular concentration of ATP ranges between 1 to 10 mM (Beis and Newsholme, 1975), and ~0.1% of this reservoir (Schwiebert, 2001) may theoretically be released into the extracellular space (up to 10 μmol/L) of the renal microenvironment in PKD, which includes the interior of encapsulated cysts (as in ADPKD) (Schwiebert, 2001), the renal interstitial space and/or the nephron lumen. The mechanisms of the extracellular release could hypothetically involve (Jacobson and Boeynaems, 2010): (1) apoptosis and necrosis of cystic epithelial cells (CECs) as well as destruction of normal renal epithelial cells with disease progression (Goilav, 2011); (2) non-lytic mechanisms (Bowler et al., 2001) involving CECs, that requires the exocytosis of secretary granules, vesicular transport and membrane channels and include ATP-binding cassette transporters, pannexins and connexins (Lohman et al., 2012), possibly in response to mechanical stretch [as shown in bladder epithelial cells (Ferguson et al., 1997)], hypoxia (Bergfeld and Forrester, 1992), dysfunction of cystoproteins (Schwiebert et al., 2002), increased cellular metabolism (Wilson, 1997; Sullivan et al., 1998) and other ATP release mechanisms that may be abnormal in PKD (Schwiebert et al., 2002); and (3) release of ATP from other local sources such as infiltrating inflammatory cells and renal nerves (Bailey et al., 2000).

Once in the extracellular fluid, ATP is capable of activating purinergic receptors, which are one of the most abundant receptor families in mammalian tissues (Abbracchio et al., 2009). There are two groups of ATP-responsive (P2) receptors: (i) P2Y receptors are G-protein coupled which act through a second messenger, and respond to a wide variety of nucleotides (ATP, ADP, UTP, UDP and UDP-glucose) They consist of eight members (subtypes 1, 2, 4, 6, 11, 12, 13, and 14, which vary depending on the type of G protein involved and specificity of the ligand) (Jacobson and Boeynaems, 2010) and modulate intracellular calcium and cAMP; and (ii) P2X receptors are rapidly acting non-selective cation channels that are calcium permeable, and which open primarily after binding to ATP (Schwiebert, 2001). They consist of eight subtypes, and in response to ATP-ligand binding, cause plasma membrane depolarization which allows calcium influx from external stores (Schwiebert, 2001).

Extracellular ATP is rapidly degraded in the plasma (Gorman et al., 2007) and in order for it to have a physiological or pathological function, it requires accumulation in the local microenvironment. The net build-up of ATP in PKD is likely to be dependent on a number of factors (Di Virgilio, 2012): (i) the pattern of P2 receptor expression in cystic and non-cystic renal tissue as well as in infiltrating inflammatory cells; (ii) the level of expression of nucleotide hydrolyzing enzymes (CD39 and CD73), which breakdown ATP and generate adenosine, which has anti-inflammatory and anti-tumor effects (Di Virgilio, 2012).

## Evidence for ATP release into the local microenvironment and P2 receptor alteration in PKD

Fluid extracted from micro-dissected cysts from ADPKD patients was found to contain pharmacologically relevant levels of ATP (up to 10 μ M) (Wilson et al., 1999), which is a thousand times higher than that found in peripheral blood (Lazarowski et al., 2001). Primary cultures of renal epithelial cells from ADPKD and ARPKD patients release between two to five times more ATP, both across the apical and basolateral membrane, under resting conditions and with hypotonic challenge, compared to non-ADPKD cells (Wilson et al., 1999; Schwiebert et al., 2002). This might, in part, be due to the slower degradation of ATP in ADPKD cells due to a reduced expression of CD39 (Xu et al., 2009) or other mechanisms. ATP, in concentrations of 4 nM, was also detectable in cyst fluid from *cpk* mice (a murine model that phenotypically resembles ARPKD, and is due to a mutation in *cystin*) (Hillman et al., 2004). However, the presence of ATP in the interstitial space, nephron lumen or urine of patients and animals with PKD has not been reported and is not known. In the microenvironment of tumors (which are thought to share some analogy to the pathogenesis of PKD) (Ghiringhelli et al., 2009), ATP has been shown to accumulate, and under these circumstances, thought to be primarily due to increased cancer cell metabolism and tumor-associated inflammation (Ghiringhelli et al., 2009; Di Virgilio, 2012). This could potentially also be the case in PKD.

Several studies have examined the expression of P2 receptors *in vitro* and *in vivo* in the cyst lining epithelium in PKD. In primary cultures of ADPKD and ARPKD cells from humans, mRNA for both P2X and P2Y receptors was found to be expressed (Xu et al., 2009). In *cpk* mice, immunohistology showed that collecting duct cysts were positive for P2X_7_ protein (Hillman et al., 2002). Similarly, in human ARPKD, P2X_7_ was also expressed in the epithelial cells of dilated collecting ducts and cysts but not present in normal human fetal kidneys (Hillman et al., 2004). In the Han:Sprd rat model of PKD, CECs from heterozygous rats were positive for P2Y (Y_2_ and Y_6_), P2X_5_ and P2X_7_ (Turner et al., 2004). Taken together, these data suggest that ATP accumulates in the PKD microenvironment, and that CECs possess the appropriate receptors to be able to respond to the presence of the ligand.

## Ciliary dysfunction in PKD alters the autocrine ATP-P2 axis, intracellular calcium and chloride secretion in cystic epithelial cells

Growing evidence suggests that extracellular ATP has a physiological role in maintaining the health and function of renal epithelial cells, and that this is dependent on an intact and functioning cilium. In immortalized normal human kidney tubular epithelial cells (HK-2 cell line), P2X_7_ receptors were localized to the primary cilia, supporting the possibility that ATP-P2 signaling is involved in mechanosensation and cyst formation (Chang and Ong, 2013). In addition, in *C. Elegans*, ATP synthase (which produces ATP from ADP) physically associates with the polycystins, LOV-1 and PKD2, suggesting that ATP synthase activity could be dysfunctional in PKD and might be a factor contributing to extracellular accumulation of ATP (Hu and Barr, 2005). The importance of a functioning cilia for ATP-purinergic signaling was further revealed from *in vitro* studies of collecting duct principal cells from Oak Ridge Polycystic kidney disease (orpk^tg737^) mice [a model of ARPKD in which cells lack a cilium due to a mutation in *polaris* (Hovater et al., 2008)]. These studies showed that the complete absence of an apical cilia impairs ATP secretion in response to mechanical, chemical and osmotic stimuli, in collecting duct principal cells (Hovater et al., 2008).

In normal renal epithelial cells extracellular ATP promotes chloride secretion and regulates intracellular calcium (Wildman et al., 2003). In PKD mutant cells, accumulating evidence suggests that chloride secretion in response to ATP is exacerbated. For example, over-expression of the cytoplasmic COOH-terminus of PC-1 in mouse cortical collecting duct cells prolonged the duration of chloride conductance in response to ATP, supporting the hypothesis that PKD mutant cells are more sensitive to some of the physiological effects of ATP (Hooper et al., 2003; Wildman et al., 2003). Therefore, it has been hypothesized that the dysfunction of the PC signal transduction pathways promotes the “secretagogue activity” of extracellular ATP by stimulating a calcium-activated chloride channel *via* purinergic receptors (Hooper et al., 2003; Wildman et al., 2003). In addition to chloride, ATP also acts in an autocrine fashion to stimulate intracellular calcium *via* purinergic receptor signaling. However, the intracellular calcium response to ATP is impaired in both murine and human ADPKD cells (Hovater et al., 2008; Xu et al., 2009). The latter was also associated with a reduced expression P2X_7_ and CD39 (Xu et al., 2009).

## Functional evidence suggesting that extracellular ATP-P2 signaling directly promotes cyst expansion and interstitial inflammation in PKD

### Cyst expansion (chloride-driven fluid secretion and proliferation of CECs)

In normal inner medullary collecting duct cells extracellular purinergic agonists can be mitogenic or co-stimulatory with other growth factors (Ishikawa et al., 1997). Therefore, in PKD, ATP has been postulated to have a role in the expansion of encapsulated renal cysts (as in ADPKD) or cystic tubular expansion (as in the ARPKD). It has been suggested that because cysts in the former are “encapsulated,” ATP accumulation may have a more important pathogenic role because of sequestration within the cyst interior, but this has not been proven (Schwiebert, 2001).

To date the functional roles of ATP-P2 signaling on the mechanisms of cyst expansion has been examined *in vitro* in cultured cells and in a zebrafish model. Collectively, these studies have indicated that ATP-P2 signaling may either accelerate or attenuate cyst growth. Similar to normal tubular epithelial cells (Kishore et al., 1995, 2000), in primary cultures of human PKD cells, ATP agonists (ATP, Bz-ATP and UTP) increased intracellular calcium levels and secretory anion transport by activating chloride-dependent secretion (that was independent of cAMP/PKA/CFTR) (Schwiebert et al., 2002). In contrast, Hillman and colleagues examined the role of ATP-P2 signaling in the initial steps of cyst formation, by growing CEC aggregates *ex vivo* from *cpk* mice (Hillman et al., 2004). In this study, the exposure of these cells to 2'- and 3'-*O*-(4-benzoylbenozoyl)-adensosine 5'-triphosphate (BzATP, a P2X_7_ agonist) reduced the number of cysts that formed by approximately one-third (Hillman et al., 2004). Cyst size and proliferation was not altered but there was a non-significant increase in caspase-3 activity (Lazarowski et al., 2001). Exogenous ATP and UTP also reduced cyst number in this model but to a much lesser extent (nearly 10%) (Lazarowski et al., 2001). In comparison to *cpk* mice CECs, in MDCK cell-derived cysts, treatment with non-specific P2 receptor antagonists (Reactive Blue 2, suramin) or removal of ATP from culture medium with apyrase (by 50%) attenuated the cAMP-ERK-dependant growth by ~50% (Turner et al., 2007). In this model, cyst growth was not affected by treatment with a non-selective P2X inhibitor, Coomassie Brilliant Blue G), suggesting a role for P2Y receptors (Turner et al., 2007). Similarly, in a separate report, in MDCK cysts derived from principal cells (Clone C7), ATP-dependent cyst growth was driven by fluid secretion rather than cell proliferation and was synergistic with cAMP (Buchholz et al., 2011). The latter was largely dependent on extracellular ATP and attenuated by the P2 receptor antagonist, suramin (Buchholz et al., 2011), and the effects were not evident in MDCK cells derived from intercalated cells (Buchholz et al., 2011). Consistent with the data in MDCK cells, in a zebrafish model of ADPKD (morpholino induced knockdown of *Pkd2*), a P2X_7_ antagonist (oxidized ATP) markedly reduced the cystic dilatation and peritubular oedema in pronephric ducts compared to the control (no treatment) or to a P2X_7_ agonist (Bz-ATP) (Chang et al., 2011). This was associated with a reduction in cell proliferation and p-ERK activity (Chang et al., 2011).

The contrasting results mentioned above are most likely due to differences in the experimental design (particularly the stage of cyst growth), the cellular model and the type of PKD examined. Perhaps, ATP-P2 signaling reduces cyst *formation* (as in *cpk* CEC aggregates) but promotes expansion once the cysts have actually formed. Further *in vivo* data using genetically orthologous models of PKD are awaiting to provide clarification regarding the role of ATP-P2 signaling in initiation and growth of cysts in PKD.

### Renal interstitial inflammation

Renal interstitial inflammation is recognized as an important factor in the progression in PKD (Ta et al., 2013). Macrophage accumulation may promote cyst growth and interstitial inflammation, and be detrimental to the progression of PKD (Swenson-Fields et al., 2013; Ta et al., 2013). The release of excess extracellular ATP is a “danger signal” that is likely to lead to interstitial inflammation *via* inflammatory signaling pathways (Idzko et al., 2007; Ivison et al., 2011). To date, the role of ATP-P2 signaling in mediating interstitial inflammation associated with PKD has not been examined. Based on data from other experimental models, one might hypothesize that extracellular ATP (released into the renal interstitium by inflammatory cells and CECs) could have a proinflammatory effect (Deplano et al., 2013). For example, in antibody-mediated autoimmune glomerulonephritis, renal injury, glomerular macrophage accumulation and the urinary excretion of the monocyte chemoattractant protein-1 was attenuated by P2X_7_ deficiency (in mice) or by administration of a P2X_7_ antagonist (A-438079) (in rats) (Taylor et al., 2009) Similarly, in a murine model of renal interstitial fibrosis (unilateral ureteral obstruction), interstitial macrophage and myofibroblast accumulation, interstitial fibrosis and tubular cell apoptosis was also attenuated by P2X_7_ deficiency (Goncalves et al., 2006). Lastly, preliminary data shows that treatment of rats with unilateral ureteral obstruction with a P2X_7_ antagonist (Brilliant Blue G) also attenuated interstitial inflammation and fibrosis but increased tubule cell proliferation (Leite et al., 2012). The latter raises the possibility that the ATP-P2 system could have complex and divergent effects *in vivo* in PKD.

## Future directions and potential for ATP-P2 signaling in the therapy of human PKD

Clinical trials (Phase 1 and 2) to determine the safety and efficacy of P2X_7_ receptor antagonists in chronic inflammatory diseases (rheumatoid arthritis, inflammatory bowel disease) are presently in progress [reviewed in Arulkumaran et al. (2011)]. In PKD, further preclinical evidence is required before clinical trials of P2X_7_ receptor antagonists can be considered. Experiments comparing disease progression in compound/double knockout mice using P2X^−/−^_7_ mice and genetically orthologous/non-orthologous murine models of PKD are required (Hillman et al., 2005). In addition, in experimental models of cancer, the over-expression of either CD39 or CD73 promotes tumor progression due to loss of tumor-associated inflammation (Synnestvedt et al., 2002; Eltzschig et al., 2009). On the other hand, in PKD, the loss of interstitial inflammation due to the transgenic expression of CD39 or CD73, might be renoprotective, as shown in experimental renal ischaemia (Crikis et al., 2010). Studies to determine the effects small molecule inhibitors (such as Brilliant Blue G, suramin, A-438079 or preferably those used in clinical trials) during the early as well as the established phases of PKD in small animal models are also needed. In particular, the stage- and disease-specific effects (relevant to human disease) of ATP-purinergic signaling in preclinical models needs to be understood (Schwiebert et al., 2002). The effects of natural and existing small molecule modulators of ATP-P2 signaling in the pathogenesis of PKD should not be forgotten, as this may accelerate translation to the clinic. For example, the methylxanthine, caffeine, induced ATP release in smooth muscle cells (Katsuragi et al., 2008), and it is possible that effects on P2 receptors underlie its ability to promote chloride driven fluid secretion in human ADPKD cells (Belibi et al., 2002). Similarly, the effects of existing pharmacological inhibitors, such as clopidogrel and ticlopidine which affect P2Y_12_ receptors (Savi and Herbert, 2005), on cyst growth in *in vitro* models, could be screened.

## Conclusion

**Figure 1:**
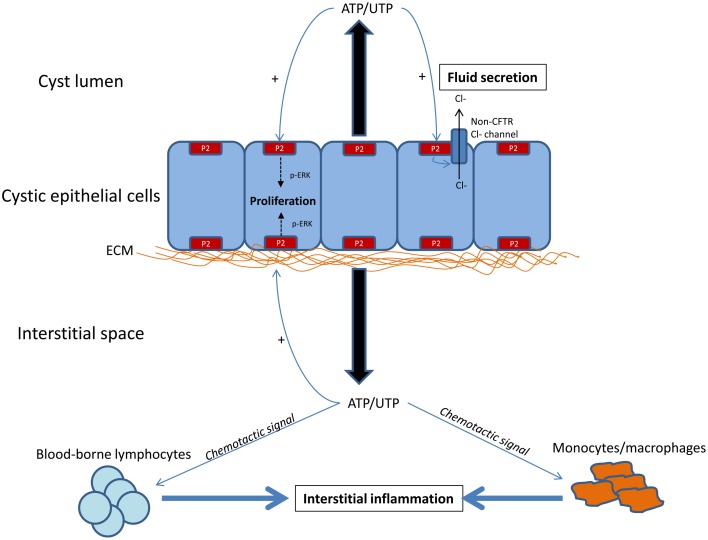
**Hypothetical diagram showing the potential role of ATP-P2 signaling in the pathogenesis of cyst growth and interstitial inflammation in PKD**. Current data suggests that extracellular ATP is increased in the microenvironment of the kidney affected by PKD, due to apical/basolateral release by cystic epithelial cells. Reduced degradation or increased cellular sensitivity to ATP may also contribute to net accumulation of ATP in the local environment. The presence of extracellular ATP has paracrine and autocrine effects on numerous cells, including cystic epithelial cells and interstitial inflammatory cells. In cystic epithelial cells, *in vitro* data suggests that P2 receptor activation promotes chloride-driven fluid secretion (independent of PKA-cAMP-CFTR mechanism) and proliferation (via p-ERK), both of which lead to cyst growth. In addition, preclinical data from non-PKD animal models suggests that P2 signaling promotes the chemotaxis of macrophages and lymphocytes, promoting interstitial inflammation. However, the hierarchy by which ATP-P2 signaling mediates proliferation, chloride secretion and interstitial inflammation in PKD is not known. In addition, the localization of P2 receptors (basolateral vs. apical distribution) or whether specific receptors have unique functions is not known.

Evidence accumulated to date supports that ATP-P2 signaling is potentially important in the pathogenesis of PKD. However, the study of ATP-P2 signaling in PKD is presently in an early phase of investigation (Figure 1) and further work, particularly preclinical *in vivo* studies, are needed before randomized controlled trials using small molecular inhibitors of P2 receptor antagonists in humans with PKD can be considered.

### Conflict of interest statement

The author declares that the research was conducted in the absence of any commercial or financial relationships that could be construed as a potential conflict of interest.
